# Caffeine Protects Dopaminergic Neurons From Dopamine-Induced Neurodegeneration via Synergistic Adenosine-Dopamine D2-Like Receptor Interactions in Transgenic *Caenorhabditis elegans*

**DOI:** 10.3389/fnins.2018.00137

**Published:** 2018-03-07

**Authors:** Rafael V. M. Manalo, Paul M. B. Medina

**Affiliations:** Biological Models Laboratory, Department of Biochemistry and Molecular Biology, College of Medicine, University of the Philippines Manila, Manila, Philippines

**Keywords:** caffeine, dopaminergic neurons, adenosine receptor, neurodegeneration, Parkinson's disease, DOP2R, neuroprotection

## Abstract

Previous studies have suggested that caffeine reduces the risk of L-DOPA-induced dyskinesia. However, caffeine is also known to promote dopamine signaling, which seemingly contradicts this observed effect. To this end, the study aimed to clarify the mechanism of caffeine neuroprotection *in vivo* when excess dopamine is present. Transgenic *Caenorhabditis elegans* (UA57) overproducing dopamine was exposed to caffeine for 7 days and monitored by observing GFP-tagged dopaminergic (DA) neurons via fluorescence microscopy. Caffeine (10 mM) prevented neuronal cell loss in 96% of DA neurons, with a mean GFP intensity that is 40% higher than control (0.1% DMSO). To confirm if cAMP plays a role in the observed neuroprotection by caffeine, cAMP levels were elevated via forskolin (10 μM), an adenylyl cyclase activator. Forskolin (10 μM) exposure did not confer neuroprotection and was similar to control (0.1% DMSO) at the 7th day, suggesting that cAMP is not the sole secondary messenger utilized. Rotigotine (160 μM), a dopamine D2-like receptor (DOP2R) agonist, was not able to confer significant neuroprotection to the nematodes. This suggests that DOP2R activation is necessary but insufficient to mimic neuroprotection by caffeine. Lastly, co-administration of caffeine (10 mM) with olanzapine (160 μM), a DOP2R antagonist, eliminated neuroprotection. This suggests that the protective effect must involve both adenosine receptor antagonism and activation of DOP2Rs. Taken together, we show that caffeine protects DA neurons from dopamine-induced neurodegeneration and acts by modulating adenosine receptor-DOP2R interactions in *C. elegans*.

## Introduction

Caffeine is the most widely used psychoactive drug. It is an integral part of day-to-day life, due in part to its effects on alertness and cognition that are of prime importance in many human activities. Previous studies have shown the benefits of taking caffeine on memory and learning, as well as in the retardation of cognitive decline (Ritchie et al., 2007). For the most part, its enhancing effects are attributed to the antagonism of adenosine A2A receptors (A2ARs)—ligands of which, like adenosine, bind to induce sleepiness—which may explain the psychostimulatory effects of caffeine (Ferré, 2008a). In addition, adenosine A2AR antagonism has been implicated in several pathways against neurodegeneration, many of which are suggestive of protection against Parkinson's disease. For instance, caffeine has been shown to protect dopaminergic neurons against toxins such as 1-methyl-4-phenyl-1,2,3,6-tetrahydropyridine (MPTP), and ablation of A2ARs led to the loss of this neuroprotective effect (Chen et al., 2001; Xu et al., 2010, 2016; Bagga and Patei, 2016). This finding was supported by the fact that L-3,4-dihydroxyphenylalanine (L-DOPA) administration in adenosine A1 receptor (A1AR), A2AR, or double A1A-A2A knockout mice reduced the incidence of L-DOPA-induced dyskinesia (LID) (Xiao et al., 2011). Through these, it was shown that antagonism or deletion of adenosine receptors, particularly A2ARs, modulated the effects of dopamine. Indeed, retrospective studies as well as some clinical trials have shown that people who consumed caffeinated drinks reduced their risk of developing LID, and from thereon caffeine was regarded as a potential neuroprotective agent against Parkinson's disease (PD)—especially benefitting motor control (Postuma et al., 2012; Wills et al., 2013). However, caffeine is a known psychostimulant, and studies have also shown that it increases dopamine signaling by actively interacting with dopaminergic (DA) neurons in *Drosophila melanogaster* (Nall et al., 2016). To this end, a paradox exists: caffeine improves dopamine signaling but also prevents the negative effects of excessive dopamine signaling. In an effort to further the development of caffeine-based drugs for treatment of PD, an understanding of the mechanisms that rationalize these two effects must be investigated.

Of great advantage to this study is the availability of various strains of *Caenorhabditis elegans* to assess neurodegenerative phenomena. These nematodes have a translucent body lining, which allows real-time monitoring of neurons as opposed to most vertebrate models that require sacrifice for dissection and consequent immunocytochemistry. Further, many transgenic strains are available which express human proteins associated with neurodegenerative disorders. Therefore, the simultaneous use of different strains expressing different proteins allows researchers to more validly correlate results with present human experience. For instance, *C. elegans* has been used to investigate the innate immune response against proteins tightly linked to amyotrophic lateral sclerosis (ALS), a neurodegenerative, arguably inflammatory disease (Vérièpe et al., 2015). Drug discovery for preventing amyloid-β and tau aggregation or hyperphosphorylation in Alzheimer's disease (AD) has also been done in previous years (Brandt et al., 2009; Fatouros et al., 2012; Alexander et al., 2014; Manalo et al., 2017). The presence of genes for neurodegenerative diseases that have human orthologs, such as amyloid precursor protein (APP), tau, and UNC13A, as well as the wide array of transgenic strains, constitute the formidable advantage of using *C. elegans* to study neurodegeneration (Link, 2006; Wolozin et al., 2011; Alexander et al., 2014).

In this study, we show that caffeine protects DA neurons from neurodegeneration induced by excessive biosynthesis of endogenous L-DOPA. Further, we show that caffeine acts by synergizing dopamine D2-like receptor (DOP2R) activation with antagonism of a putative adenosine receptor (A_R_) ortholog. We show that either DOP2R agonism or A_R_ antagonism is necessary but insufficient in conferring neuroprotection, supporting previous findings. Lastly, we attempt to solve the paradox of dopamine signaling by proposing DOP2R availability as an alternative, coherent explanation for increased dopamine signaling in *C. elegans*.

## Materials and methods

### Preparation of nematode plates

For this study, nematodes in OP50-seeded nematode growth media (NGM) plates were prepared according to the protocol of Steirnagle (1999), with slight modifications. Briefly, 0.375 g NaCl, 2.125 g agar, and 0.3125 g bactopeptone were dissolved in 125 mL of dH_2_O, mixed and autoclaved for ~15–20 min at 121°C. The flask was cooled to touching temperature (~60°C), after which 125 μL each of 1 M CaCl_2_, 5 mg/mL cholesterol in 70% EtOH and 1M MgSO_4_ were added, followed by 3.125 mL of 1M KPO_4_. The solution was mixed vigorously, and was then poured into petri plates (Merck, 47 mm) using sterile techniques. After the NGM hardened, 100 μL of *E. coli* strain OP50 in aqueous solution was added. For each treatment group, 20 *C. elegans* strain UA57 nematodes at stage L4 to young adulthood were worm-picked onto each plate. *C. elegans* is a free-living, non-parasitic nematode, while the *E. coli* strain OP50 is non-pathogenic; thus, all procedures were done in accordance with the precautions for Biosafety Level 1.

### Fluorescence microsocopy of *C. elegans* strain UA57

For this study, the following treatments were administered: DMSO (0.1% v/v), forskolin (10 μM), caffeine (10 mM), rotigotine (160 μM), and olanzapine+caffeine (160 μM). For olanzapine+caffeine, 320 μM of olanzapine was mixed with 20 mM caffeine in a volume ratio of 1:1, to obtain final olanzapine and caffeine concentrations of 160 μM and 10 mM, respectively. The concentration of caffeine was chosen based on a previous study comparing caffeine concentrations from 5 to 100 mM, showing optimal worm lifespan extension delay of age-associated paralysis at 10 mM caffeine (Sutphin et al., 2012). The concentration of forskolin was based on a study showing forskolin-induced cAMP accumulation via adenosine A_2A_Rs at 10 μM concentration (Florio et al., 1999). Meanwhile, the olanzapine concentration was based on a study on antipsychotic drugs showing complete elimination of dauer formation and significant decrease in the lifespan of *C. elegans* using olanzapine (160 μM)—implying its full inhibitory effect at the said concentration (Weeks et al., 2010). From here, the concentration of rotigotine (160 μM) was chosen, since rotigotine and olanzapine are known dopamine D2-like receptor (DOP2R) agonists and antagonists, respectively. Nematodes were exposed to each treatment for a minimum of 2 h, after which the DA neurons were observed via fluorescence microscopy (Evos® FL) and counted as Day 0. For all observations, green fluorescent protein (GFP) intensity was set at 40%, with 10x magnification. Succeeding observations were obtained at days 3, 5, and 7, and were analyzed using ImageJ. The percentage of intact neurons out of the four cephalic and two anterior deirid neurons, as well as the minimum/maximum GFP intensity per neuron and average GFP intensity were then obtained and graphed as mean ± standard error of measurement (SEM). Neurons were considered lost when either the absence of fluorescence or the presence of small round bodies was observed. Blebbing neurons were not considered as lost neurons to minimize errors in scoring, as blebbing may at times be difficult to distinguish from intact neurons, and is not necessarily irreversible (Tang et al., 2012).

### Sudan black staining of lipid stores in *C. elegans*

To monitor changes in lipid stores, fixed Sudan Black staining was performed on *C. elegans* according to the protocol of Barros et al. (2012), with slight modifications. Briefly, 100 nematodes at L4 stage were worm-picked per treatment group (forskolin and caffeine) and were allowed to stand for 7 days. On the 7th day, nematodes were collected with 1 M KPO_4_ into 1.5 mL eppendorf tubes. The nematodes were allowed to stand for 20 min, with most supernatant discarded. This washing was then repeated at least once more, with a final volume of at least 100 μL. Then, 400 μL of 1M KPO_4_ was added, followed by 500 μL of 2X Modified Ruvkun's Witches Brew (160 mM KCl, 60 mM NaCl, 14 mM Na_2_EDTA, 1 mM spermidine, 0.2% β-mercaptoethanol, and 2% formaldehyde), after which the solution was incubated at ~25°C for 1 h on a rotator. After settling and three rinses of 1M KPO_4_, the volume was reduced to 100 μL followed by 300 μL of 1M KPO_4_ and 600 μL of 2-propanol. The solution was allowed to stand for 15 min and ~0.9–1.0 μL of supernatant was taken from the solution depending on the remaining supernatant—taking care to leave ~100 μL of supernatant in which the nematodes are understood to be immersed in. Sudan black (16 mg/mL in 70% EtOH, 1 mL) was then added and the suspension was incubated at room temperature overnight on a rotator. The following day, black supernatant was drained via micropipetting, after allowing the suspension to settle. The precipitates were transferred to a clear NGM plate, and nematodes were viewed for fix-stained lipid stores via bright-field microscopy (10x).

### Statistical analyses

The effect of each treatment group per day were analyzed and generally compared with one another for variations via the one-way analysis of variance (ANOVA). To determine the difference between each treatment group, a *post-hoc* Bonferroni-Holm method was employed to correct for errors due to multiple comparisons. For all statistical analyses, “^*^” denotes significance at *p* < 0.05. For all graphs, the mean of each data set was plotted with its respective standard error of measurement (mean ± SEM).

## Results

### Caffeine protects dopaminergic neurons in CAT-2 overexpressed transgenic *C. elegans*

**Figure 1:**
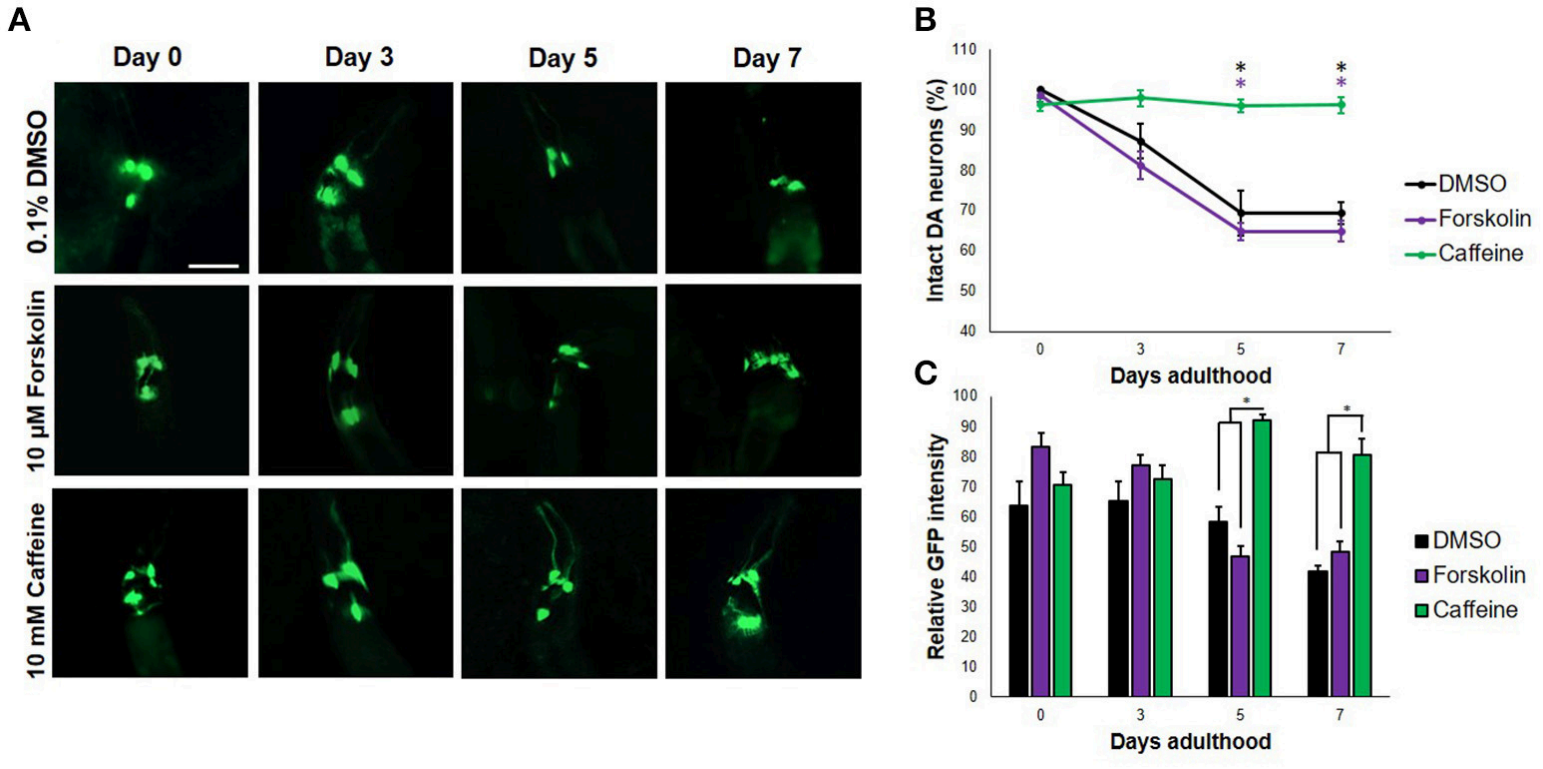
Caffeine protects DA neurons from dopamine-induced neurodegeneration**. (A)** Transgenic *C. elegans* expressing GFP tagged to DA neurons and overexpressing CAT-2 was treated with vehicle (0.1% DMSO), forskolin (10 μM), or caffeine (10 mM) and was followed-up for 7 days via fluorescence microscopy. Images were analyzed using ImageJ. **(B)** Caffeine significantly protected 96% of DA neurons for 7 days post-adulthood, while forskolin was essentially as ineffective as vehicle. **(C)** The average GFP intensity was 40 and 48% higher than forskolin and control at day 7, respectively. ^*^ significance at *p* < 0.05. Statistical analyses were performed using one-way ANOVA followed by a *post-hoc* Bonferroni-Holm method for multiple comparisons.

When transgenic *C. elegans* nematodes were exposed to forskolin (10 μM) and caffeine (10 mM), caffeine preserved DA neurons consistently for a span of 7 days (Figures 1A,B). In particular, both the four cephalic and two anterior deirid neurons were protected. In contrast, cephalic neurons degenerated in forskolin, with complete cell loss of the anterior deirid neurons. Since neurodegeneration in the control group (0.1% DMSO) followed a similar trend as in forskolin, neurodegeneration was attributed to excess dopamine due to the overexpressed CAT-2 enzyme. Significant difference in neuron count was observed at day 7 (*p* < 0.05), with caffeine having an average GFP intensity comparable with previous days. Overall, caffeine-exposed *C. elegans* DA neurons were noted to have been protected and showed a brighter average GFP intensity at the end of 7 days as compared with forskolin- and DMSO-exposed nematodes (Figures 1A,C).

**Figure 2:**
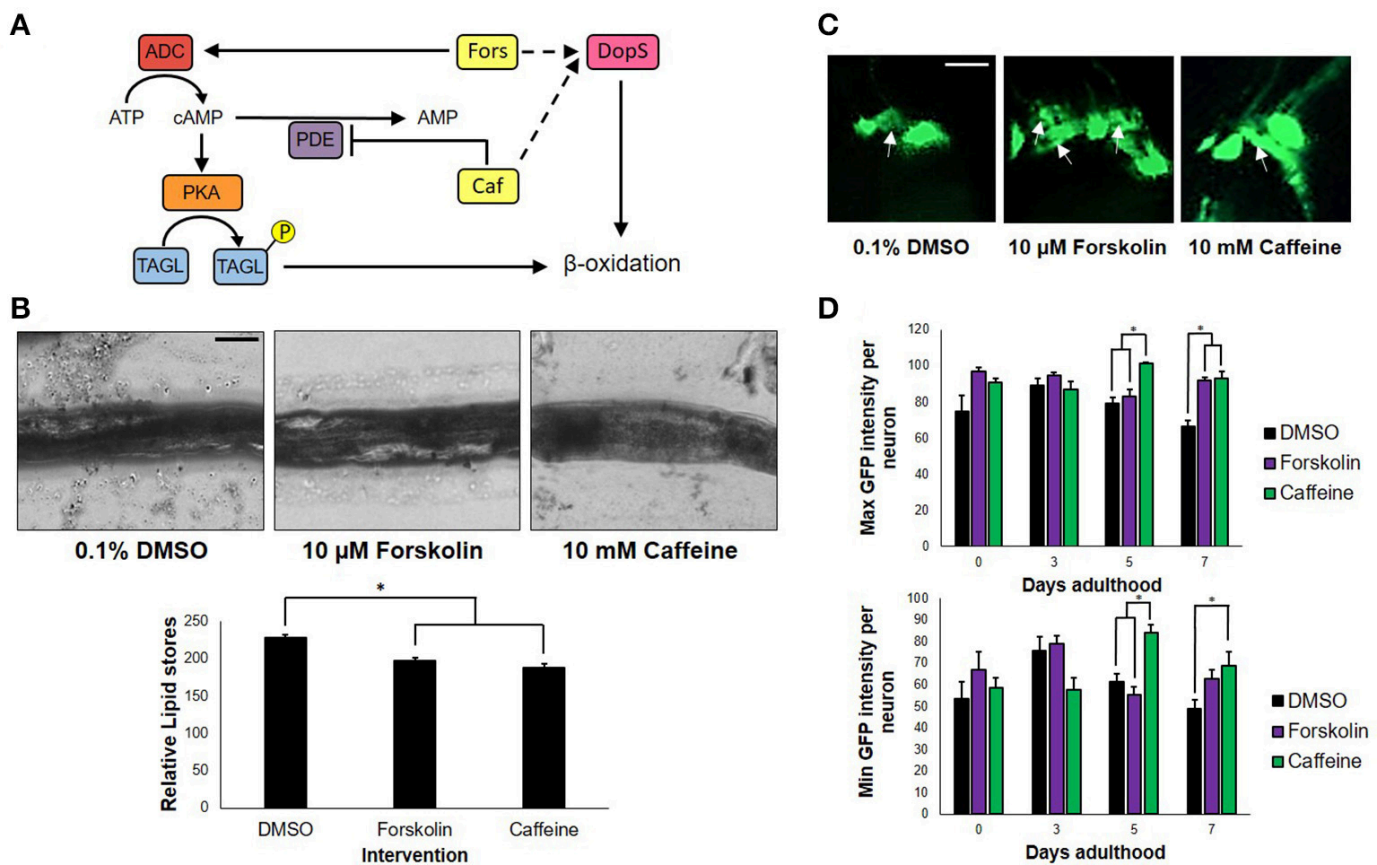
cAMP is not the sole secondary messenger utilized by caffeine. **(A)** Forskolin and caffeine, an adenylyl cyclase activator, and phosphodiesterase inhibitor, respectively, both increase intracellular cAMP levels and promote β-oxidation. **(B)** Lipid stores were markedly lower between vehicle and treatment, and were essentially the same in foskolin and caffeine. Images were analyzed using ImageJ. **(C)** Caffeine showed enhanced DA neuron survival with less instances of cell blebbing. White arrows indicate neurons in the blebbing stage of apoptosis at 7 days post-adulthood. **(D)** The maximum and minimum GFP intensities per neuron are essentially the same, indicating that the increased GFP intensity of caffeine is a product of cell survival and not an increased expression of GFP alone. Images were analyzed using ImageJ. ^*^ significance at *p* < 0.05. Statistical analyses were performed using one-way ANOVA followed by a *post-hoc* Bonferroni-Holm method for multiple comparisons.

To determine whether or not the concentrations of forskolin and caffeine were comparable, ~100 nematodes each were exposed to both treatments and control for 7 days, which were then sacrificed for fixed staining using Sudan Black. Less intense stains were observed for the forskolin- and caffeine- treated groups, which were also essentially the same in intensity (Figure 2B). Since both caffeine and forskolin act to promote lipolysis and subsequent beta-oxidation of lipid stores through promotion of cAMP signaling, this result suggests that the concentrations used for both treatment groups were essentially comparable in terms of cAMP potentiation (Figure 2A). However, the fact that forskolin was not able to mimic neuroprotection conferred by caffeine implies that cAMP signaling alone does not play a major role in the observed effect. Further, we noted that DA neurons had less instances of apoptosis when exposed to caffeine, compared with forskolin and control (Figure 2C). This suggests that exposure to caffeine does confer neuroprotection, and not merely an increase in the expression of GFP. We hypothesized that the neuroprotection in *C. elegans* was mediated by adenosine receptor antagonism unique only to caffeine. It is possible, therefore, that the cAMP-mediated effect of forskolin *in vivo* was not as effective due to the lack of a G protein-coupled receptor (GPCR) interaction. While significant differences in the maximum and minimum GFP intensities per neuron exposed to caffeine and forskolin were observed at days 5 and 7, the results imply that both forskolin and caffeine significantly increase GFP intensity compared with vehicle, but the difference between forskolin and caffeine may not readily be observed *in vivo*. These imply, at least, that the brighter average GFP intensity (Figure 1C) could be induced by elevated cAMP levels (Figure 2D).

### DOP2R agonism is necessary but insufficient in caffeine-induced neuroprotection

**Figure 3:**
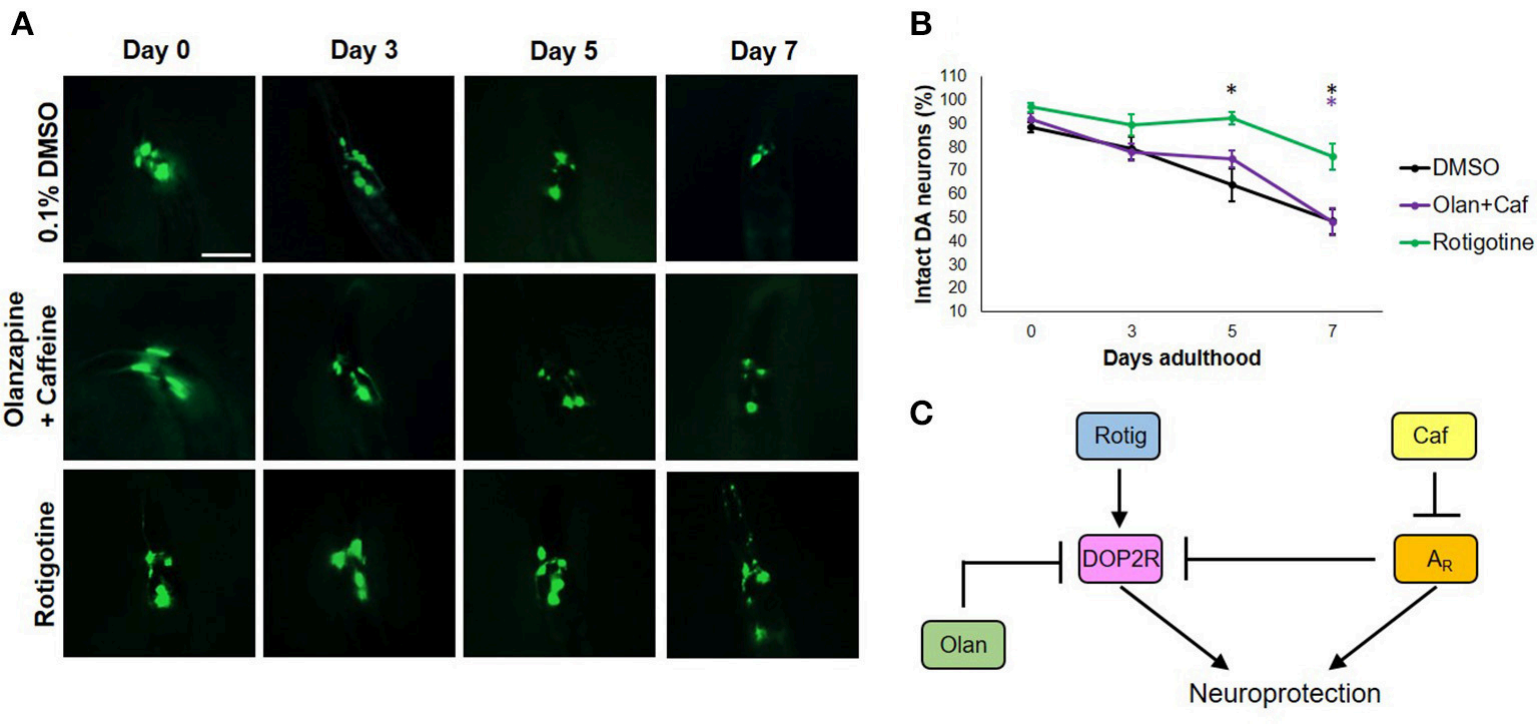
Caffeine confers neuroprotection by modulating A_R_s and DOP2Rs. **(A)** Transgenic *C. elegans* expressing GFP tagged to DA neurons and overexpressing CAT-2 was treated with vehicle (0.1% DMSO), rotigotine (10 μM), or olanzapine+caffeine (10 mM) and was followed-up for 7 days via fluorescence microscopy. Images were analyzed using ImageJ. **(B)** Olanzapine significantly eliminated neuroprotection by caffeine, while rotigotine protected neurons up to day 5, but was not able to mimic neuroprotection on day 7. **(C)** Caffeine acts by antagonizing adenosine receptors (A_R_s) which by so doing increases the availability of DOP2Rs that together confer an enhanced neuroprotection. Neither DOP2R agonism (rotigotine) nor A_R_ antagonism (olanzapine+caffeine) alone can confer neuroprotection when excess dopamine is present. ^*^ significance at *p* < 0.05. Statistical analyses were performed using one-way ANOVA followed by a *post-hoc* Bonferroni-Holm method for multiple comparisons.

To get an insight on the interactions between dopamine and adenosine receptors in *C. elegans*, we used rotigotine (160 μM) and olanzapine (160 μM) to agonize and antagonize dopamine D2-like receptors (DOP2Rs), respectively. Interestingly, rotigotine treatment led to a prognosis similar to caffeine (Figure 3B). However, all six neurons essentially preserved until day 5 began to significantly degenerate at day 7 (Figures 3A,B). In contrast, olanzapine ablated caffeine-induced neuroprotection, being non-significant compared to control (*p* < 0.05). Even if significance was found between nematodes exposed to rotigotine and olanzapine + caffeine, it is unlikely to be biologically relevant as evidenced by the comparable percentages and error bars. In addition, since rotigotine is an agonist of DOP2Rs, these results suggest that DOP2R agonism is not sufficient to mimic the level of protection conferred by caffeine. Therefore, DOP2R activation is necessary but insufficient in mimicking caffeine-induced neuroprotection. On the other hand, olanzapine antagonizes DOP2Rs. When caffeine was administered with olanzapine, the protection seen previously was lost. Since the combination leads to antagonism of both putative adenosine and dopamine D2-like receptors, results suggest that neither DOP2R activation alone nor adenosine receptor antagonism alone is sufficient to mimic caffeine-induced neuroprotection, and it is likely that adenosine receptors interact with DOP2Rs when antagonized. These results support an interaction seen in higher vertebrate models, where adenosine receptors normally antagonize DOP2Rs. Therefore, administration of caffeine may increase DOP2R availability, which is an effect countered by olanzapine (Figure 3C). Further, this suggests the presence of a putative adenosine receptor ortholog in *C. elegans*—a receptor type known to antagonize DOP2Rs, which was consistent with previous suggestions from candidate genes (Hobert, 2005; Sutphin et al., 2012).

## Discussion

### Caffeine does not induce dopamine biosynthesis in *C. elegans*

As shown previously, caffeine protected DA neurons against degeneration promoted by excessive dopamine. This finding implies that caffeine does not increase dopamine synthesis—because if it did, then the observed neurodegeneration would in fact worsen. The first issue is whether or not caffeine acts in a similar manner in *C. elegans* as with higher animal models. Typically, caffeine works by antagonizing all types of A_R_s (Ribeiro and Sebastião, 2010), with a markedly high affinity for A1 and A2A receptors (Chou and Vickroy, 2003). A1 A_R_s are suppressive in nature, and are known to oppose neuronal excitation; On the other hand, A2A A_R_s are known to utilize cAMP as a second messenger, along with some types of voltage-gated Ca^2+^ channels (Fredholm et al., 1994). Thus, caffeine action on both receptors would promote neuronal excitability and prevent its opposition by the binding of adenosine to A2A—a mechanism tightly associated with sleep promotion (Satoh et al., 1998). This is not, however, the solely proposed mechanism. In one study, it was shown that caffeine—by antagonizing A_R_s—sensitized dopamine D2 receptors normally co-localized with them in the rat brain, leading to contralateral rotational behavior after exposure to 6-hydroxydopamine (6-OHDA). In this way, they proposed that caffeine may in fact worsen dopamine-related neurotoxicity and motor dysfunction (Pollack et al., 2010). Further, the pathological rotational behavior in rats was also suggestive of possible depletion of brain dopamine, which parallels to neurodegeneration (Willis and Sandyk, 1993). However, given that caffeine was shown to protect neurons (Kolahdouzan and Hamadeh, 2017), enhance cognition and physical performance (Cappelletti et al., 2015), and that caffeine can reduce aberrant motor symptoms found commonly in parkinsonism (Roshan et al., 2016), a more profound mechanism must be present. To this end, suggestions were made that it is in fact the antagonism of A1 A_R_s that enhances locomotion and provides for central nervous stimulation (by antagonizing the suppressive A1 A_R_s), and that it is the antagonism of A2A A_R_s that lead to dopamine receptor sensitization and therefore aggravation of dopamine-related symptoms (Hadfield and Milio, 1989; Popoli et al., 1996; Górska and Golembiowska, 2015). This way, the locomotion and cognition-enhancing effects of caffeine are rationalized, while maintaining its limitation as a drug against dopamine-induced neurodegeneration. For instance, co-administration of caffeine with MDMA was found to aggravate toxic extracellular dopamine levels (Górska and Golembiowska, 2015). However, this mechanism along with those aforementioned do not explain the protective effects of caffeine against nigostriatal damage by 6-OHDA, as well as protection against L-DOPA and MPTP-induced motor impairments that can be mimicked by A2A A_R_ antagonists but not antagonists of A1 A_R_s (Blandini, 2003).

To this end, the neuroprotective and locomotion-enhancing effects of caffeine likely lies in its antagonism of A2A A_R_s, contrary to many previous reports. With knowledge that caffeine promotes dopamine signaling (Nall et al., 2016), however, a seeming contradiction arises. How can caffeine both protect against dopamine-induced neurodegeneration and motor impairment, and promote dopamine signaling at the same time? This question requires much clarification. Our results with caffeine administered to transgenic nematodes over-producing dopamine showed protection and prevention of cell body loss in 96% of DA neurons (Figure 1). Therefore, it is not likely for caffeine to promote endogenous dopamine levels, which is counter-productive to neuroprotection in this context. It is possible that instead of inducing dopamine biosynthesis, caffeine would either (1) induce dopamine receptor expression, or (2) increase the availability of DOP2Rs, to promote dopamine signaling. This hypothesis is not unlikely, and does in fact support a previous finding in humans (Volkow et al., 2015), where caffeine was found to increase dopamine D2/D3 receptor availability. As to whether or not adenosine receptor orthologs are antagonized by caffeine in *C. elegans*, adenosine has been previously shown to antagonize caffeine-induced lifespan extension in *C. elegans*—pointing out to a conserved caffeine-A_R_ interaction in the nematode model (Bridi et al., 2015).

### Caffeine protects DA neurons through the A_R_ and DOP2R interactions

**Figure 4:**
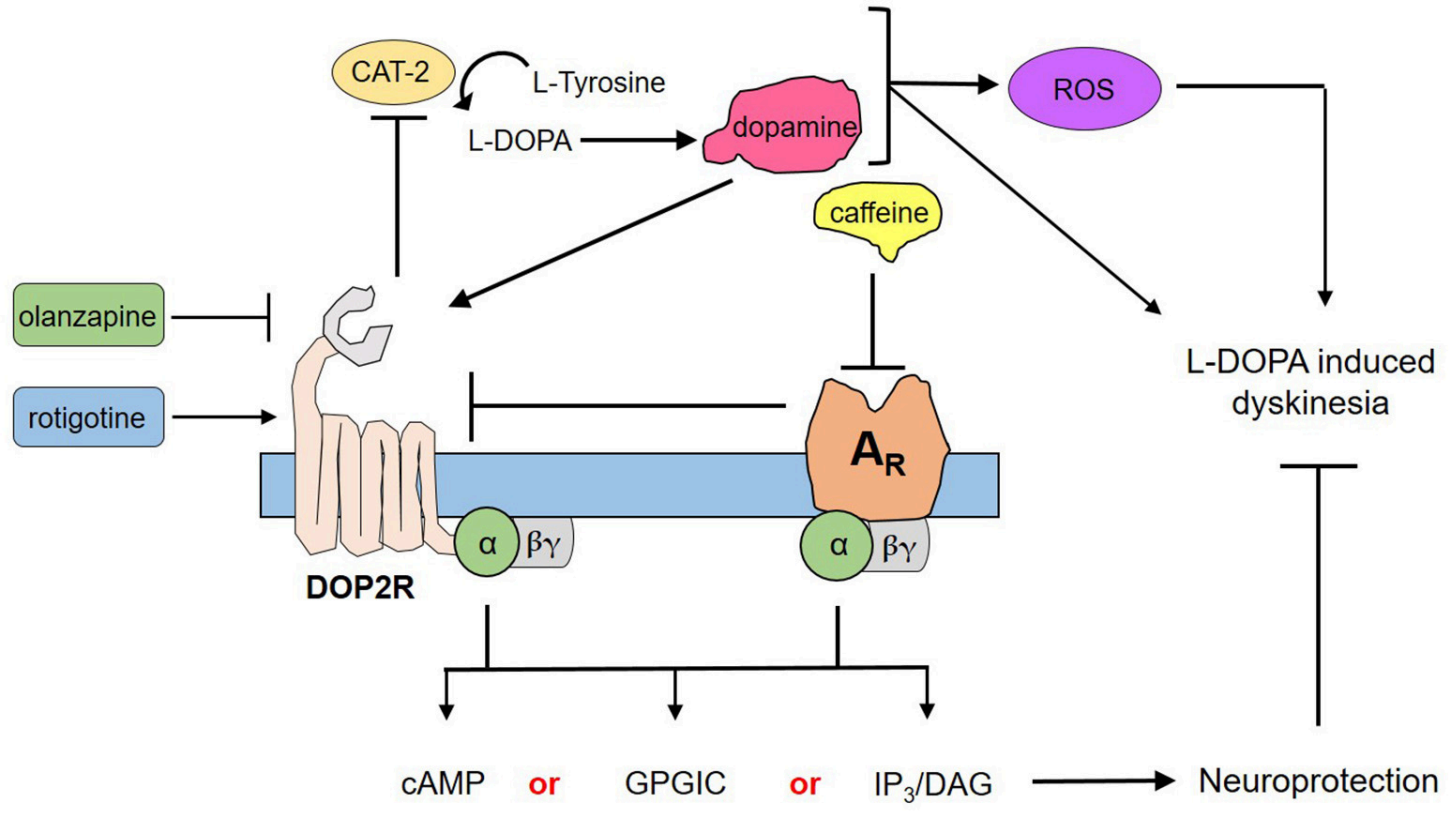
Proposed mechanism for the neuroprotection of caffeine against dopamine-induced neurodegeneration. Transgenic *C. elegans* UA57 overexpresses CAT-2, which is a rate-limiting enzyme in dopamine biosynthesis. Its synthesis and degradation produces reactive oxygen species (ROS), which in excess could damage DA neurons. Meanwhile, its action on dopamine receptors can mediate dopamine-related pathological behaviors. Caffeine acts by antagonizing A_R_s, which increases the availability on presynaptic DOP2Rs. DOP2Rs are autoreceptors and are naturally inhibitory; hence, upon activation it would reduce the synthesis and release of dopamine. Both the action on A_R_s and DOP2Rs would elicit secondary messengers that are either combinations of cyclic adenosine monophosphate (cAMP) and G protein-gated ion channels (GPGIC), cAMP and inositol triphosphate/diacylglycerol (IP_3_/DAG), or all three—which together with less ROS and less dopamine could confer neuroprotection to UA57 and account for the alleviation of L-DOPA induced dyskinesia.

Through the use of forskolin, rotigotine, and olanzapine, we have elucidated a proposed mechanism by which caffeine acts to confer neuroprotection to DA neurons (Figure 4). Briefly, forskolin activates both adenylyl cyclase and CAT-2—producing cAMP that has been shown to mediate pathways leading to neuroprotection as a second messenger. However, forskolin was not able to mimic neuroprotection by caffeine, suggesting that cAMP is not the sole secondary messenger in action, and may in fact involve other pathways such as G protein-coupled ion channels or the IP_3_/DAG pathway, among others (Dascal, 2001; Tuteja, 2009). Could DOP2Rs then be the sole player in this neuroprotection? This question was answered by the findings on rotigotine, which is a known DOP2R agonist (Figure 3). In the study, rotigotine was not able to fully mimic caffeine-induced neuroprotection, suggesting the insufficiency of DOP2R agonism. When caffeine is administered with olanzapine, neuroprotection is lost. Since olanzapine antagonizes DOP2Rs, this finding implies that caffeine acts in part by activating DOP2Rs. However, since activating DOP2Rs via rotigotine was insufficient, it is rational to propose that caffeine indirectly modulates DOP2Rs by binding a different receptor. We therefore hypothesize that it binds to a putative adenosine receptor in *C. elegans* that is similar to human adenosine A2A. This hypothesis is in fact supported by previous findings of candidate genes for adenosine receptors in *C. elegans* (Hobert, 2005), as well as the known interactions between A2AR and DOP2R in higher vertebrate models (Fuxe et al., 2005; Ferré, 2008b). Further, these results imply that the known action of caffeine to improve dopamine signaling works by antagonizing the putative adenosine A2A ortholog, which by so doing increases the availability of DOP2Rs to provide more binding sites for dopamine. Lastly, modulation of putative adenosine A2A and DOP2R leads to separate downstream cascades that may synergistically confer neuroprotection, as suggested by the non-efficacy of adenosine receptor antagonism or DOP2R activation alone. Hence, caffeine confers neuroprotection by the combined efforts of putative adenosine A2A receptors and DOP2Rs.

If the mechanism is as such, then consistent with previous reports, caffeine sensitizes DOP2Rs. However, this again brings into question the possible aggravation of dopamine-induced neurodegeneration as was previously pointed out. To note, such aggravation is realistic, and caffeine as aforementioned was found to promote rotational behavior suggestive of pathological coupling with dopamine. However, several studies have also pointed out a turning event in relation to chronic exposure. When rats were chronically treated with caffeine solution, they became tolerant to turning induced by caffeine as well as by xanthine analogs such as theophylline (Garrett and Holtzman, 1995). Further, it was shown that in chronic exposure, tolerance to caffeine-induced rotational behavior as well as extinction of L-DOPA-induced rotation would be observed, such that co-administration would result to altered L-DOPA responses (Yu et al., 2006). This suggests that in chronic exposure to caffeine, dopamine-induced pathological behavior such as rotation in rats improves. In some cases, caffeine may enhance rotational behavior as shown by the same author (Yu et al., 2006), which was said to contrast with the attenuation of L-DOPA-induced rotation upon deletion of A2A A_R_ genes (Freduzzi et al., 2002). Instead of being paradoxical, this points out to the dose dependence of caffeine action, which was repeatedly shown to be beneficial at low doses and toxic at high doses (Bridi et al., 2015; Al-Amin et al., 2016). In the context of our proposed mechanism in *C. elegans*, therefore, a low-dose administration of caffeine (10 mM) could increase availability of DOP2Rs to dopamine and enhance the dopamine response, which would then modulate neuroprotection. The key concept in this mechanism is the autoreceptor function of DOP2Rs. These receptors, primarily found in DA neurons as presynaptic receptors, control both the rate and amount of dopamine release by DA neurons—the absence of which could lead to Parkinson's disease and schizophrenia (Tepper et al., 1997; Ford, 2014) Hence, when dopamine is formed from endogenous L-DOPA in the transgenic strain, some of it activates presynaptic DOP2Rs—resulting to less dopamine, either by reducing dopamine biosynthesis or dopamine release. This cycle of DOP2R activation both in control and rotigotine-treated *C. elegans* could not protect against neurotoxicity, as dopamine is overproduced. Caffeine exposure might therefore protect neurons by (1) antagonizing A_R_ orthologs thereby sensitizing DOP2Rs and enhancing their inhibitory action on dopamine release, and (2) modulating downstream cascades leading to neuroprotection. Hence, DOP2R action is due in part to its nature as an autoreceptor (Figure 4).

### Caffeine as a pharmacological drug against dopamine-induced neurotoxicity

The results of this study contradict implications of several previous reports, and sufficiently support others. Studies in previous decades have pointed out on the synergism of caffeine and dopaminergic interventions in sensitizing dopamine receptors and aggravating toxic dopamine release—implying its non-usage in this regard. Further, previous reports suggested that caffeine increases brain dopamine levels (Strömberg and Waldeck, 1973)—a concept that we attempt to correct in this paper. In more recent years, reports have shown the beneficial effects of caffeine administration, both prophylactic and therapeutic, in improving dopamine-related symptoms, such as L-DOPA-induced dyskinesia and other parkinsonian disorders (Ross et al., 2000; Schwarzchild, 2012). Therefore, contrary to intuition, caffeine is a drug that both improves dopamine signaling and reduces dopamine-induced neurotoxicity and eventual neurodegeneration. In other words, caffeine is able to promote bodily processes mediated by dopamine, the most important of which is movement in this study, while protecting neurons from the consequence of its overproduction. This mechanism, as we propose, is modulated by DOP2Rs and A_R_s—acting in synergism to both promote and protect. Many studies have implied the role of caffeine in neuroprotection, albeit through the use of coffee extracts or participants who have previously taken coffee (Ross et al., 2000; Wills et al., 2013). However, it is also important to note that coffee contains several compounds other than caffeine, which by themselves have also been shown to confer neuroprotection (Lee et al., 2013; Huber et al., 2015). In fact, even decaffeinated coffee can provide neuroprotection in *Drosophila* (Trinh et al., 2010). In an effort to isolate the effects of caffeine in this context and to contribute significantly to pharmacologic data, we utilized pure caffeine in this study. Further, we studied neuroprotection in an effort to consolidate previous data on the locomotion-enhancing properties of caffeine and its supposed neuroprotection of motion-regulating neurons. Indeed, this study has shown, to our knowledge, the first proof of caffeine neuroprotection in a neurodegenerative model of *C. elegans* employing dopamine overproduction. This has many consequences. For one, this implies the possibly beneficial effects of caffeine against addiction-related pathology like neurodegeneration, cognition decline and reward circuitry—all of which are mediated by dopamine in addictive drugs. Secondly, this revives the possible role of caffeine as an effective adjuvant to L-DOPA in reducing adverse events, which eventually points out to the determination of its effective concentration to maximize benefit and minimize adverse events in PD.

## Conclusion

In this study, we show that caffeine (10 mM) protects 96% of DA neurons against dopamine-induced neurodegeneration in transgenic *C. elegans* UA57. Determination of lipid stores via fixed Sudan Black staining showed lower levels of lipids in forskolin (10 μM)- and caffeine-treated nematodes, which were markedly similar to each other. This suggests that the cAMP potentiation of forskolin and caffeine was essentially the same in the concentrations used. Since forskolin was not able to confer neuroprotection, we propose that cAMP is not the sole second messenger being utilized by caffeine. Administration of rotigotine (160 μM) did not confer neuroprotection to *C. elegans*—indicating that mere agonism of DOP2Rs is not sufficient. Co-administration of olanzapine with caffeine eliminated neuroprotection—suggesting that both DOP2Rs and A_R_s are necessary to confer neuroprotection, but neither are sufficient on their own. We therefore show for the first time that caffeine does not increase dopamine synthesis but increases the availability of DOP2Rs in *C. elegans*, and that its binding to putative A_R_ orthologs communicates with DOP2Rs to regulate dopamine overproduction and confer neuroprotection against induced neurodegeneration, through second messengers other than cAMP.

## Author contributions

All authors listed have made a substantial, direct and intellectual contribution to the work, and approved it for publication.

### Conflict of interest statement

The authors declare that the research was conducted in the absence of any commercial or financial relationships that could be construed as a potential conflict of interest.
